# Coronary Angiography Print: An Automated Accurate Hidden Biometric Method Based on Filtered Local Binary Pattern Using Coronary Angiography Images

**DOI:** 10.3390/jpm11101000

**Published:** 2021-10-01

**Authors:** Mehmet Ali Kobat, Turker Tuncer

**Affiliations:** 1 Department of Cardiology, Firat University Hospital, Firat University, Elazig 23119, Turkey; 2 Department of Digital Forensics Engineering, College of Technology, Firat University, Elazig 23119, Turkey; turkertuncer@firat.edu.tr

**Keywords:** coronary angiography print, hidden biometric, filtered LBP, NCA, biometrics

## Abstract

Background and purpose: Biometrics is a commonly studied research issue for both biomedical engineering and forensics sciences. Besides, the purpose of hidden biometrics is to discover hidden biometrics features. This work aims to demonstrate the biometric identification ability of coronary angiography images. Material and method: A new coronary angiography images database was collected to develop an automatic identification model. The used database was collected from 51 subjects and contains 2156 images. The developed model has to preprocess; feature generation using local binary pattern; feature selection with neighborhood component analysis; and classification phases. In the preprocessing phase; image rotations; median filter; Gaussian filter; and speckle noise addition functions have been used to generate filtered images. A multileveled extractor is presented using local binary pattern and maximum pooling together. The generated features are fed to neighborhood component analysis and the selected features are classified using k nearest neighbor classifier. Results: The presented angiography image identification method attained 99.86% classification accuracy on the collected database. Conclusions: The obtained findings demonstrate that the angiography images can be utilized as biometric identification. Moreover, we discover a new hidden biometric feature using coronary angiography images and name of this hidden biometric is coronary angiography print.

## 1. Introduction

A coronary angiogram is one of the X-ray imaging techniques [1,2]. Coronary angiography is the gold standard diagnostic method of coronary artery disease (CAD) and CAD is the disease that causes the most mortality worldwide. Although coronary angiography is generally used in the diagnosis of coronary artery disease, postmortem coronary angiography is performed to investigate the cause of death and to determine the coronary anatomy [3,4,5,6,7,8].

Dr. Mason Sones invented coronary artery imaging on 30th October 1958 and he injected a contrast material into the right coronary artery of a person and he imaged coronary arteries. It was the first application of invasive cardiology [9]. After that, coronary angiography has been used frequently not only as an imaging method but also as a treatment method [10,11].

In 2009 and 2010, 260,995 and 335,113 coronary angiography operations were performed respectively. In these years (2009 and 2010), total population of Turkey was 71 and 72 million, consecutively [12]. With the rapid increase in centers where coronary angiography is performed and the population increasing, it can be thought that much more coronary angiography is performed today. Coronary angiography was performed on 9360/per million people in Germany in 2017 [13]. Therefore, there are variable coronary angiography images in the medical centers and these images should be used with machine learning models to create smart cardiology applications.

CT angiography can be used instead of invasive coronary angiography to evaluate anatomy thanks to advanced tomography devices. In a different study, biometrics with a CT angiograph can also be planned.

The study area in which previously unknown features are used to identify individuals is called hidden biometry [14,15,16]. Hidden biometrics is more robust against possible forgery than visual biometrics [17]. Many studies are performed on hidden biometrics by using machine learning techniques in the literature [18,19,20,21]. However, it has been observed in the literature that studies on hidden biometrics are less than visual biometrics. 

Some of the studies on hidden biometrics in the literature are presented below. 

Nait-Ali [17] presented a hidden biometric study and features of visible and hidden biometrics were presented in their study. In particular, medical images were evaluated using magnetic resonance (MR) and Hand /Lung X-Ray images on hidden biometrics, and different areas of use were highlighted. In another study, Nait-Ali [16] evaluated the use of biomedical signals and images in security applications. This evaluation is on the use of parameters obtained from medical data in hidden biometrics. This study confirms that the medical images used in hidden biometrics are robust to forgery. Rida et al. [22] proposed a hidden biometrics approach for emotion prediction. The main purpose of their study is to perform emotion prediction with four motions (waving, drinking, clapping, and throwing) of a subject. For this purpose, regression and classification were performed. Maheshwari and Choudhary [23] presented an approach for hidden biometrics. The main purpose of this study is to analyze brain MR images and CT scans for medical diagnosis. This method was based on artificial brain transformation. Kulkarni et al. [24] applied a local binary pattern for authentication. Their method performs finger vein biometric identification. In their study, 50 subjects were used and they obtained a new database using finger vein samples. They performed denoising and enhancing processes to the collected finger veins and features were extracted using the local binary pattern (LBP). They attained 94.34% accuracy on their collected database using the LBP model. Kabbara et al. [25] applied a phalanx segmentation method for biometric identification. In their study, X-Ray images were used for experimental results. 88 right hand XRI was obtained from 42 subjects. Fourier descriptors were utilized for feature extraction. They achieved 98.75% true identification rate. Lozhnikov et al. [26] presented a hidden biometric identifier method using signature. The main purpose of this study is to protect electronic text documents and paper. Therefore, a digital watermark was embedded on the text to protect the originality of digital and paper documents.

In this study, a new hidden biometric identification method is proposed using coronary angiography images. In the proposed method, it was determined that coronary angiography images achieved successful results in human identification. The obtained angiography image database was collected from the Firat University Cardiology department. Angiography images are one of the variants of X-ray images. By using the coronary angiography images, artery structures of the heart have been analyzed, and many coronary artery diseases have been diagnosed. People with normal coronary anatomy between the ages of 30–60 were included in this research. Philips 7220261131 medical systems (Nederland B.V. Veenpluis 4-6 5684 PC Best, Turkey) device was used to gather coronary angiography. Angiography videos were gathered from this machine, and these videos are framed, and angiography images were obtained. The collected angiography image database contains 2156 images of 51 subjects. The size of these images is 512 × 512 pixels, and they are stored in JPEG format. We used healthy subjects. 

Our main motivations are given as follows. The coronary angiography images have been used to diagnose coronary artery disorders. However, we realized that the heart anatomies are unique by examining the coronary artery images. Therefore, a study using artificial intelligence was planned to determine whether the coronary anatomy of each person is really different. Personal identification/biometrics studies such as fingerprints and retinal scans encouraged us to identify coronary artery anatomy. The aim of our study is in special cases where identification is necessary; In cases where it is difficult to identify the person after burns and after serious accidents, postmortem coronary angiography can be used to identify the person’s coronary anatomy. Since postmortem coronary angiography is a difficult process, but it can be performed in mandatory situations. Coronary angiogram images can be used not only postmortem but also in cases where high security is required in living people.

Our proposed model has two main/key contributions and they are;

- Hidden biometrics is one of the popular research issues. This work contributes to the hidden biometrics research area and we investigated new biometric features in this work. To validate the human identification feature of the coronary artery images, a novel coronary angiography image database was collected and published publicly (see Section 2).
- An accurate image classification model is presented using an LBP feature extractor. LBP is an effective feature generator and it has commonly been used in biometrics methods. To increase the feature extraction capability of the LBP, a multileveled and filtered extractor is presented. Moreover, NCA chooses the top features and kNN classifies the selected features. This model attained a 99.86% accuracy rate on the collected database. Furthermore, our proposal can be used in other image classification problems.

## 2. Materials and Methods

### 2.1. Material

**Figure 1:**
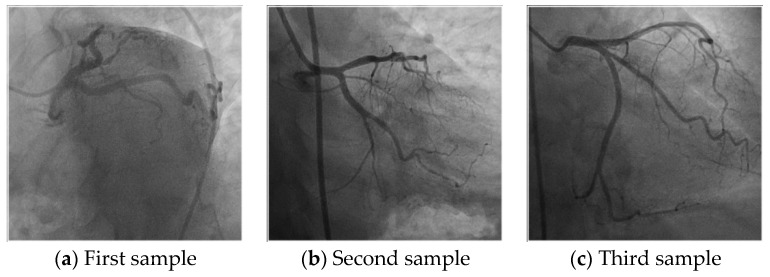
Samples image of the collected coronary angiography image database or human identification.

Initially, we collected an angiography images database to detect coronary artery disorder automatically. However, we realized that the heart anatomies are unique by examining the collected coronary angiography images. Therefore, we decided that to collect a new image database to identify humans. Herein, we used coronary angiography images and these images are collected from 51 subjects. This database contains 2156 images. This database was collected from Firat University Hospital between 2019–2021 retrospectively and it has ethical approval. The properties of the collected images are given as follows. The file format of the collected images is jpg and the size of these images is 512 × 512 pixels. 28 out of these subjects are male and 23 out of them are female. The age range of the male and female subjects are to 31 from 69 (50.92 ± 9.78) and to 31 from 80 (56.73 ± 13.52) consecutively. Sample images of this database are also denoted in Figure 1.

Furthermore, this database was published publicly and the users/researcher can download this database from https://www.kaggle.com/turkertuncer/coronary-angiography-printURL (accessed on 15 August 2021).

### 2.2. Method

**Figure 2:**
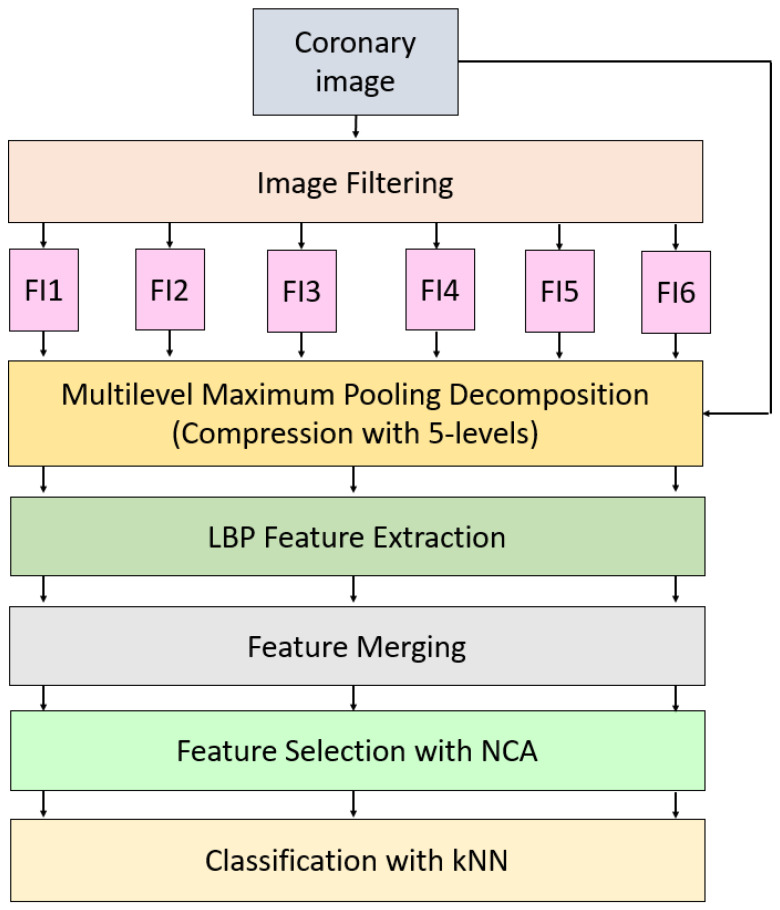
The proposed filtered images and multilevel maximum pooling-based LPB-NCA image classification model. Herein, FI1, FI2, …, FI6 denote filtered images.

This work presents a new LBP-based learning model. This work consists of Pre-processing, LBP [27], and maximum pooling-based feature generation, feature chosen based on NCA [28], and classification with kNN [29]. The main objective of this model is to attain high prediction performance on the collected coronary angiography images and improve the classification ability of the LBP-based model. In the literature, image augmentation is one of the commonly used methods and variable operators have been used to increase the number of images. We used the commonly used operator to the number of features increasing. Therefore, four operators are used to create filtered images. Moreover, compressed versions of these images are generated using maximum pooling operator. LBP extract features from all generated images and the most informative 413 features are selected by NCA. These 413 features are fed to kNN classifier and the results are obtained. Diagram of the proposed LBP-NCA based human identification model using coronary angiography images is denoted in Figure 2.

As can be seen in Figure 1, six image filtering operators have been applied to collected coronary images and these filters are speckle-noise addition, image rotation (90, 180, 270 degrees), median filtering, and Gaussian filtering. By deploying these filters, six filtering images (they are denoted in Figure 1 as FI1, FI2, …, FI6) are obtained. Multilevel maximum pooling compression is applied to six filtered images and raw/original images. This decomposition function created five compressed images from each input image. In total, 7 × 6 = 42 images (35 compressed images, 6 filtered images, and 1 original image) are utilized as input of the LBP. LBP generates 59 features from each image. The extracted/generated features by LBP are merged and 59 × 42 = 2478 features are created. The best 413 (1/6 of the merged features) out of these 2478 features are chosen by NCA. kNN is employed to classify these 413 features. The steps of the proposed model are given in below in detail.

#### 2.2.1. Preprocessing

In the preprocessing phase, image filtering and image compression operations have been implemented. 42 images are obtained for feature generation using our proposal preprocessing. The steps of this preprocessing phase are given below.

***Step 0:*** Read each coronary angiography image from the collected biometric database.

(1) $$I=Dt_{k},\;k\in \left\{1,2,\ldots ,2156\right\}$$

where I is an image with a size of 512 × 512 and Dt is the collected database with 2156 coronary angiography images.

***Step 1:*** Generate filtered images using Gaussian, median, rotation, and speckle noise filters.

(2) $$FI^{1}=rotate\left(I,90\right)$$

(3) $$FI^{2}=rotate\left(I,180\right)$$

(4) $$FI^{3}=rotate\left(I,270\right)$$

(5) $$FI^{4}=medfilt\left(I,3\times 3\right)$$

(6) $$FI^{5}=gaus\left(I\right)$$

(7) $$FI^{6}=sn\left(I,0.05\right)$$

In Equations (2)–(7), the filtered image (FI) generation is explained. Herein, four filters are used and these filters are image rotation (rotate(.,.)), median filtering (medfilt(.,.)) with 3 × 3 sized blocks, Gaussian filter (gaus(.)) and speckle noise addition (sn(.,.)) with 0.05 intensity. 

**Figure 3:**
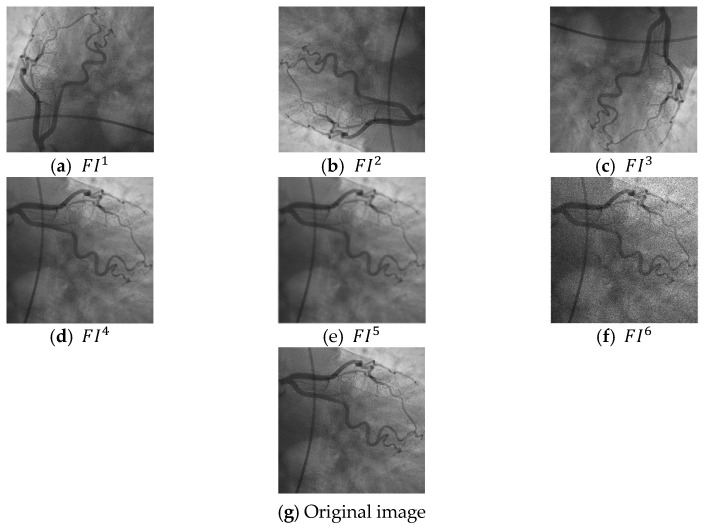
Sample filtered images and original images. (**a**) the first filtered image, (**b**) second filtered image, (**c**) third filtered image, (**d**) fourth filtered image, (**e**) fifth filtered image, (**f**) sixth filtered image, (**g**) the used original coronary angiography image.

Sample filtered images are demonstrated in Figure 3.

***Step 2:*** Create compressed images using the maximum pooling function. In here, 2 × 2 sized non-overlapping images have been used. Firstly, an image structure is created and it is named Im.

(8) $$Im^{1}=I$$

(9) $$Im^{j+1}=FI^{j},\;j\in \left\{1,2,\ldots ,6\right\}$$

After that, a five-leveled maximum pooling decomposition is applied to Im for generating compressed images (Cim). Equation (10) explains the used five-leveled maximum pooling compression method.

(10) $$Cim^{\left(j-1\right)\times 5+t}=maxp\left(Im^{j},2^{t}\times 2^{t}\right),\;t\in \left\{1,2,\ldots ,5\right\},\;j\in \left\{1,2,\ldots ,7\right\}$$

Herein, 35 Cim are generated using maxp(.,.) decomposition function. 

In this phase, 35 compressed and six filtered images are created.

#### 2.2.2. Feature Extraction Using Local Binary Pattern 

In this phase, LBP is the used fundamental feature creation function. LBP is a commonly used hand-crafted feature generator in the literature. It is good at feature extraction for biometrics methods. Moreover, LBP has simple computational complexity (O(n)) and it can be implemented easily. LBP divides images into 3 × 3 sized overlapping blocks. These blocks are simple neighborhood blocks. The neighbor pixels, center pixel, and signum function are used to generate features in this function. The Signum function is a basic compression function and a mathematical explanation of this function is given in Equation (11).

(11) $$\gamma \left(a,b\right)=\{\begin{array}{c} 1,\;a-b\geq 0\\ 0,a-b<0 \end{array}$$

where γ(.,.) signum function and a,b are input parameters of the signum function. In the LBP a is one of the neighbor pixels and b is a center pixel. In this work, we used the LBP feature extraction function of MATLAB and it generates 59 features from an image. The feature generation and concertation processes are defined below.

***Step 3:*** Generate features using the LBP (lbp(.)) feature extraction function.

(12) $$ftv^{j}=lbp\left(Im^{j}\right),\;j\in \left\{1,2,\ldots ,7\right\}$$

(13) $$ftv^{k+7}=lbp\left(Cim^{k}\right),\;k\in \left\{1,2,\ldots ,35\right\}$$

where ftv is generated feature vectors by the LBP extractor. Herein, 42 feature vectors are created.

***Step 4:*** Merge the created 42 feature vectors to obtain the last feature vector.

(14) $$lf\left(u+59\times \left(h-1\right)\right)=ftv^{h}\left(u\right),\;u\in \left\{1,2,\ldots ,59\right\},\;h\in \left\{1,2,\ldots ,42\right\}$$

Herein, lf defines the last feature vector with a length of 2478.

#### 2.2.3. Feature Selection Based on Neighborhood Component Analysis 

NCA is a good and effective feature selector and it has commonly been used to choose the most informative/valuable features from the generated feature vector. NCA creates weights from each feature and the qualified feature indexes are calculated using the created weights. The most informative features are selected using these indexes. In this work, the most valuable 413 features out of the created 2478 features are chosen. 

***Step 5:*** Choose the most valuable/meaningful 413 features from the generated 2478 features.

#### 2.2.4. Classification 

The last phase of the proposed filtered and multileveled maximum pooling-based LBP-NCA method is classification. In order to denote the success of the presented feature generation and selection methods, a simple/traditional distance-based classifier is used and this classifier is kNN [29]. The used kNN classifier is named Fine kNN on the MATLAB classification learner tool. The parameters of the used Fine kNN are given as follows. The distance metric is Euclidean, k value is 1, voting/distance weight is equal, and standardize is true. Moreover, 10-fold CV is utilized as a validation technique.

***Step 6:*** Classify the selected 413 features using the defined kNN classifier and calculate results.

## 3. Results and Discussions

The proposed LBP-NCA-based human identification method was programmed by a simple configured personal computer using MATLAB (2020b) programming environment. In order to create filtered images, imrotate, imnoise, imgaussfilt, and medifilt2 MATLAB functions were used. The maximum pooling function was created using an m file and the extract LBP Features function is used to create local features. In the feature choosing phase, fscnca function was used with solver, sgd (stochastic gradient descent), verbose and 1 parameter. MATLAB classification learner toolbox was utilized to generate codes of the Fine kNN classifier. 

To calculate the performance of the LBP-NCA-based human identification model, accuracy, precision, recall, and F1-score evaluation metrics were selected. Overall scores were denoted in Table 1.

As can be seen from Table 1, the proposed model yielded 99.86% accuracy, precision, recall, and F1-score respectively.

**Figure 4:**
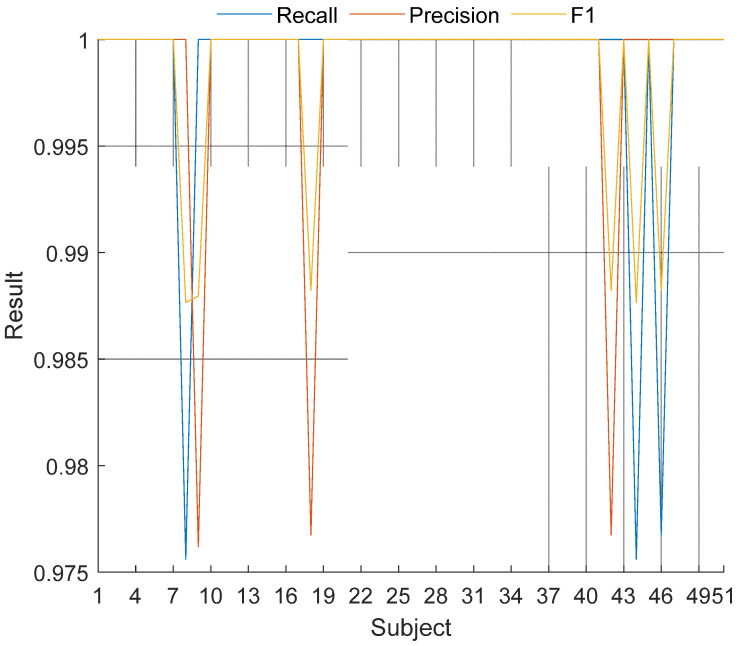
Subject-wise results of the proposed LBP-NCA-based coronary angiography print method.

Moreover, subject-wise (class-wise) results were denoted in Figure 4.

As can be seen in Figure 4, the proposed method yielded 100% recall on the 48 subjects (except for 8th, 44th and 46th subjects) and all subject-wise results are greater than 97.5%.

**Figure 5:**
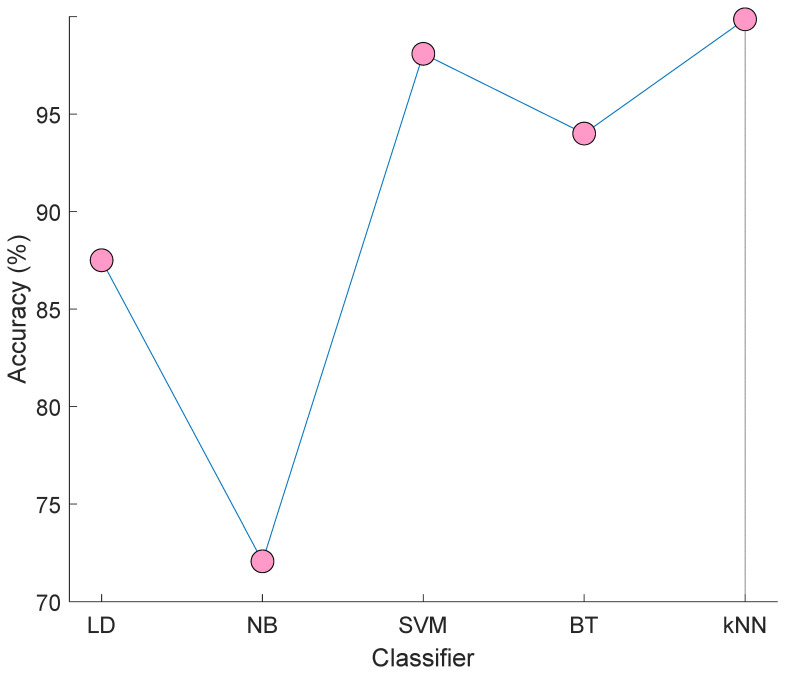
Results of the LD, NB, SVM, BT, and kNN classifiers on the selected 413 features.

Moreover, linear discriminant (LD) [30], Naïve Bayes (NB) [31], support vector machine (SVM) [32,33] and bagged tree (BT) [34] classifiers are utilized to classify the selected 413 features. Results of these classifiers and our used kNN are denoted in Figure 5.

Figure 5 demonstrated that the best classifier is kNN. Therefore, we used kNN in the classification phase. The worst classifier in NB and attained 72.07% accuracy. The best of others (except for kNN) is SVM and it yielded a 98.09% accuracy rate. By using kNN, the number of misclassified observations are calculated as three.

According to calculated results, the benefits of this research are;

- A new hidden-biometric feature is presented using coronary angiography images.
- To validate the results of the proposed coronary angiography images based on biometric identification, a new large database was collected and it is published publicly.
- A new LBP-based human identification approach is proposed in this work and this approach resulted successfully. Our proposed model can extract features at a high level by using filters and multi-level maximum pooling decomposition. By only using the LBP extractor, low-level features are extracted. Herein, we used a filtered-LBP to create features at a high level. Moreover, we have used an effective feature selector (NCA) to increase the performance of the proposed model.
- The proposed filtered LBP-NCA-based model yielded 99.86% classification accuracy.
- The robustness of the proposed is denoted using a 10-fold CV.
- By using a simple/traditional classifier, excellent results are obtained.
- The presented coronary angiography print can be used to identify humans in forensics applications. It is a new biometric feature. Therefore, there is no attack for this biometric authentication model. In this view, it is the safest model to validate human identification.
- Coronary artery imaging is a diagnostic method that can be done invasively and non-invasively. It can be used without using fingerprints, palm prints, iris recognition, and face recognition due to severe cheeks or traumas that are not accepted by the advanced practice. Again, this biometric recognition system can be used for unidentified dead IDs for the same authentication.
- We are the first team to present a biometric method using craniological images as far as we known.

Limitations

- Bigger databases can be collected in the near future and our model can be tested on the collected bigger coronary angiography images databases.
- This model is the first model. Therefore, we cannot compare other state-of-art methods to our proposal. However, our model is the reference model in the literature.
- Our proposed coronary angiography print is a new hidden biometry model. However, coronary angiography is an invasive method, it can be used in very special cases for specific biometric identification.
- Coronary artery anatomy shows changes in some cases such as occlusion due to atherosclerosis or coronary bypass surgery. However, it also shows changes due to injury or some diseases in the retinal or palmar arteries, so each method used has certain limitations. The development of coronary artery disease in individuals and related changes in anatomy may limit the use of coronary arteries for biometric. This is one of the limitations of our study.

## 4. Conclusions

Nowadays, many attacks have been applied a biometric authentication system. Therefore, hidden biometrics is a popular research issue for information security. This work presents a new hidden biometric feature and the name of it is coronary angiography print. A new coronary angiography image database was collected to deploy a new effective automatic human identification model. Our proposed identification model uses filtered images, five leveled maximum pooling decomposition, LBP feature extractor, NCA selector, and kNN classifier. The suggested model yielded 99.86% classification accuracy. The calculated results and findings frankly demonstrate the human identification ability of the coronary angiography images. 

In near future, more efficient deep learning and hand-modeled methods can be integrated into the presented model and a more effective learning method can be presented. Moreover, this method can be tested on the bigger image databases. By using a bigger image database and an effective learning model, coronary angiography images based on new generation human identification applications can be developed and these applications can be used in the forensics departments.

## Figures and Tables

**Table:** 

Metric	Accuracy	Precision	Recall	F1
Result	99.86	99.86	99.86	99.86

**Table 1:** Overall performance results (%) of the proposed LBP-NCA based model.

Metric	Accuracy	Precision	Recall	F1
Result	99.86	99.86	99.86	99.86

## Data Availability

All data generated or analyzed during this study are included in this published article.
